# Immunotherapy for pleural mesothelioma: from innovation to the frontlines of core clinical questions

**DOI:** 10.1007/s12672-025-04200-9

**Published:** 2025-12-04

**Authors:** Tatsuya Yoshida, Kozo Kuribayashi

**Affiliations:** 1 Department of Thoracic Oncology/Experimental Therapeutics, National Cancer Centre Hospital, Tokyo, Japan; 2 Department of Respiratory Medicine and Hematology, Hyogo Medical University, 1-1, Mukogawa-cho, Nishinomiya, 663-8501 Hyogo Japan

## Abstract

In August 2018, Japan’s Pharmaceuticals and Medical Devices Agency (PMDA) became the first in the world to approve immune checkpoint inhibitor (ICI) monotherapy with nivolumab for previously treated, unresectable, advanced, or recurrent pleural mesothelioma (PM), setting a global precedent. Subsequently, in October 2020, the U.S. Food and Drug Administration (FDA) approved the combination of nivolumab and ipilimumab (CheckMate 743 trial) as the first-line treatment for unresectable PM. This marked the first update to standard first-line therapy in approximately 15 years since the approval of cisplatin (CDDP) plus pemetrexed (PEM) based on the EMPHACIS trial in 2004. Most recently, in September 2024, the FDA approved pembrolizumab plus platinum and PEM (KEYNOTE-483 trial) as another first-line option for advanced or metastatic PM. These developments underscore the rapid evolution of systemic therapy for PM. However, several urgent clinical questions have emerged in real-world practice. This article aims to address these issues based on the latest evidence and expert insights.

## Introduction

Several systemic regimens for PM have now been approved by regulatory authorities worldwide:

- Cisplatin plus pemetrexed for untreated, unresectable PM (EMPHACIS trial) [1].
- Nivolumab plus ipilimumab for untreated, unresectable PM (CheckMate 743 trial) [2].
- Pembrolizumab plus platinum plus pemetrexed for untreated, unresectable PM (KEYNOTE-483 trial) [3].
- Nivolumab monotherapy for previously treated PM (MERIT trial) [4].

The advent of ICIs has revolutionized the therapeutic landscape of PM [5]. Nevertheless, several unresolved clinical questions (CQs) have arisen in real-world practice:

CQ1: Optimal selection between CheckMate 743 and KEYNOTE-483 regimens.

CQ2: Optimizing treatment strategies by histologic subtype (epithelioid vs. non-epithelioid).

Supplement: Insights from real-world evidence (RWE) following nivolumab + ipilimumab.

CQ3: Treatment options after progression on CheckMate 743.

CQ4: Treatment options after progression on KEYNOTE-483.

Supplement: Do racial or ethnic differences influence outcomes of combination immunotherapy?

CQ5: Is PD-L1 expression a reliable biomarker for treatment selection?

This article reviews the distinct characteristics of ICI–ICI and ICI–chemotherapy combination therapies, discusses their appropriate selection for first-line use, and explores evidence-based strategies for subsequent treatments.

### CQ1

**Optimal selection between CheckMate 743 and KEYNOTE-483 regimens (ITT Focus)**.

#### Background

CheckMate 743 and KEYNOTE-483 are pivotal phase III randomized trials that established immunotherapy-based first-line options for previously untreated, unresectable PM. Although both incorporate immune checkpoint inhibition, their designs differ (dual ICI vs. chemo-immunotherapy), leading to distinct efficacy and safety profiles in the intention-to-treat (ITT) populations.

#### Efficacy in ITT populations

Overall survival (OS).

Both trials demonstrated a statistically significant OS advantage over platinum–pemetrexed. Direct cross-trial comparisons are inappropriate; each regimen should be interpreted within its own study context.

Progression-free survival (PFS) and objective response rate (ORR).

KEYNOTE-483 showed a modest but statistically significant PFS improvement versus chemotherapy, whereas CheckMate 743 did not. ORR was higher with chemo-immunotherapy; however, ORR should not be over-interpreted as a surrogate for durable benefit or symptom relief, and should be considered alongside OS and PFS.

All efficacy metrics should be reported consistently with HR, 95% CI, and p-values, and with the assessment method specified (e.g., Independent Review Committee (IRC) vs. investigator) to avoid internal inconsistencies.

A concise summary of efficacy outcomes is provided in Table 1.

#### Safety (reporting framework for ITT)

Safety should be summarized consistently by grade and type across regimens: grade ≥ 3 treatment-related adverse events (TRAEs), immune-related adverse events (irAEs), discontinuations, systemic steroids, serious adverse events (AEs) requiring hospitalization, and treatment-related deaths.

Cross-trial toxicity comparisons should be cautious and not used alone to guide regimen choice in vulnerable patients.

Key safety outcomes are also summarized in Table 1, with details of grade 3–4 TRAEs and irAEs reported consistently.

#### Clinical interpretation (ITT)

Rather than a hierarchy of efficacy, the two regimens provide complementary profiles within ITT populations: dual ICI emphasizes potential for durable survival, and chemo-immunotherapy demonstrates earlier disease control. Histology-specific considerations are addressed in CQ2, to keep CQ1 focused on the ITT perspective.

**Table 1:** Summary of key efficacy and safety outcomes in phase III trials for unresectable PM

	CheckMate 743 [2] (Nivolumab + Ipilimumab vs Chemotherapy)	KEYNOTE-483 [3] (Pembrolizumab + Platinum + Pemetrexed vs Chemotherapy)
Population	Intention-to-treat (ITT)	Intention-to-treat (ITT)
Primary Endpoint	Overall Survival (OS)	Overall Survival (OS)
Median OS (months)	18.1 vs 14.1	17.3 vs 16.1
HR (95% CI)	0.74 (0.60–0.91)	0.79 (0.63–0.99)
p value (OS)	0.002	0.037
Median PFS (months)	6.77 vs 7.20	7.13 vs 7.16
HR for PFS (95% CI)	1.00 (0.82–1.21)	0.80 (0.65–0.99)
p value (PFS)	NS	0.0372
Objective Response rate (ORR)	39.6%	52.3% (IRC-assessed)
Grade 3–4 TRAEs (%)	30 vs. 32	27 vs. 15
Serious AEs requiring hospitalization (%)	N/A	18 vs. 6
Treatment-related deaths (n)	3 vs. 1	2 vs. 1
Notable irAEs	Endocrine (13.6%), Hepatic (8.5%), Pneumonitis (4.2%)	Cytopenia, Renal dysfunction, Gastrointestinal toxicity; fewer immune-related AEs
Notes	Dual ICI improved OS; non-significant PFS likely reflects delayed immune response; notable irAEs observed.	Chemo-IO improved both OS and PFS modestly; early disease control observed; safety manageable.

1. HR values are calculated for time-to-event analyses (entire OS/PFS curves), not for medians

2. All hazard ratios are presented with 95% confidence intervals (CIs)

3. ORR values are based on independent radiologic review committee (IRC) unless otherwise specified

4. NS = not significant; ITT = intention-to-treat population

### CQ2

**Optimization of treatment strategies for epithelioid and non-epithelioid subtypes**.

#### Background

PM is histologically classified into epithelioid and non-epithelioid (sarcomatoid or biphasic) subtypes, each demonstrating distinct biological behavior, immune landscapes, and therapeutic responses. Subtype-specific immune microenvironments have emerged as key determinants of response to ICIs.

#### Differences in the immune microenvironment

The non-epithelioid subtype exhibits higher infiltration of CD8⁺ T cells and macrophages with elevated PD-L1 expression [6]. The presence of immunosuppressive M2 macrophages and Tregs may make this subtype more responsive to ICI-based therapy [7].

The epithelioid subtype shows abundant B-cell infiltration (CD20⁺ B cells) and CD4⁺ T-cell dominance [8]. PD-L1 expression is relatively low-to-intermediate, suggesting potentially limited benefit from ICI monotherapy [9].

#### Evidence from randomized trials

In CheckMate 743, dual ICI therapy (nivolumab + ipilimumab) significantly prolonged median overall survival (OS) in non-epithelioid PM (18.1 vs. 8.8 months; HR 0.46 [95% CI 0.31–0.68]).

Similarly, in KEYNOTE-483, the pembrolizumab + chemotherapy arm achieved longer OS (12.3 vs. 8.2 months; HR 0.57 [95% CI 0.36–0.89]).

Conversely, in the epithelioid subtype, OS benefit was modest and not statistically significant in either trial (CheckMate 743: 18.7 vs. 16.5 months, HR 0.86; KEYNOTE-483: 19.8 vs. 18.2 months, HR 0.89).

Nevertheless, KEYNOTE-483 demonstrated a high objective response rate (ORR 67%) in epithelioid cases, indicating potential for rapid tumor shrinkage and symptom relief. A summary of efficacy outcomes by histologic subtype is provided in Table 2.

#### Clinical implications and strategy

For non-epithelioid PM, which is immunologically “hot,” dual ICI therapy (as in CheckMate 743) is recommended to achieve durable long-term survival.

For epithelioid PM, where rapid tumor control is often necessary, chemo-immunotherapy (as in KEYNOTE-483) offers early clinical benefit with manageable safety (Grade ≥ 3 TRAEs 27% vs. 15%, serious AEs requiring hospitalization 18% vs. 6%).

#### Integrated clinical viewpoint

CheckMate 743 and KEYNOTE-483 have collectively transformed the first-line treatment paradigm of PM. Nivolumab + ipilimumab is preferred for non-epithelioid and fit patients seeking durable benefit, whereas pembrolizumab + platinum/pemetrexed is a reasonable option for epithelioid or frail patients requiring early tumor control. Personalized treatment selection should integrate histology, immune contexture, tumor burden, and clinical goals.

**CQ2 supplementary: real-world evidence and clinical role of KEYNOTE-483**.

While CheckMate 743 suggested that immunotherapy benefits mainly non-epithelioid PM, emerging real-world evidence (RWE) challenges this notion (Table 2).

- Japan (Kanayama et al., *n* = 41): Epithelioid ORR 29.6%, median PFS 11.7 months [10].
- France (Bylicki et al., *n* = 201): Epithelioid OS 21.0 months vs. non-epithelioid 14.1 months [11].
- LATAM (ImmunoMeso study, *n* = 96): ORR 42.9% (epithelioid) vs. 29.3% (non-epithelioid) [12].
- Australia (RIOMeso, *n* = 119): OS 14.5 months, PFS 6.7 months, ORR 27.9%; Grade ≥ 3 TRAEs 22% (1 L) and 30% (2 L) [13].

Collectively, these RWE data suggest that ICI therapy may also offer meaningful benefits in selected epithelioid patients in clinical practice. However, such findings should be interpreted as hypothesis-generating given the heterogeneity of RWE design and population.

KEYNOTE-483 further redefined the role of chemo-immunotherapy in epithelioid PM, demonstrating a significant ORR advantage (67% vs. 47%, *p* = 0.0003) with manageable Grade ≥ 3 TRAEs (27%, lower than 30.3% in CheckMate 743). Its mechanistic basis—chemotherapy-induced immunogenic cell death enhancing PD-1 blockade—may explain its utility in less immunogenic tumors.

**Table 2:** RWE in patients treated with nivolumab plus ipilimumab as first-line treatment

Author (Study)	No. of patients (Median age)	OS (months)	PFS (months)	ORR (%)	Safety
Kanayama et al. [10]	*N* = 41 (72 years)	NR Epi/NonEpi: NR/15.2	8.6 Epi/NonEpi: 11.7/5.8	29.3% Epi/NonEpi: 29.6/36.4%	TRAEs 68.3% Grade ≥ 3 19.5%
Bylicki et al. (The Meso-Immune [GFPC 04–2021] French EAP) [11]	*N* = 201 (Epi/NonEpi: 149/52) (75 years)	18.9 Epi/NonEpi: 21.0/14.1	6.3 Epi/NonEpi: 6.3/6.6	19.9 Epi/NonEpi: 17.5/27.1	69.3% Gradae ≥ 3 23.3%
Enrico et al. (ImmunoMeso LATAM) [12]	*N* = 96 (63 years)	22 Epi/NonEpi: 23.0/19.0	8 Epi/NonEpi: 8.0/10.0	32.3 Epi/NonEpi: 42.9/29.3	43.1% Grade ≥ 3 18.5%
McNamee et al. (RIOMeso) [13]	*N* = 119 (1st/2nd: 89/30) (72 years)	14.5 1st/2nd:14.5/15.4 Epi/NonEpi: 19.1/13.0	1st/2nd: 6.7/3.1	27.9	Grade ≥ 3 1st/2nd: 22/30%
Douma et al. (FLORA) [14]	*N* = 277 (72 years)	14.6	5.5	NE	NE

OS; Overall survival, PFS; progression free survival, ORR; overall response rate, NR; Not reached, Epi; Epithelioid, Non Epi; Non-epithelioid, NE; Not evaluable

In summary, nivolumab + ipilimumab is most appropriate for non-epithelioid PM and fit patients with durable response potential, whereas pembrolizumab + chemotherapy serves as a complementary option for epithelioid or frail cases requiring rapid disease control.

A comparative overview of trial design and clinical features is presented in Table 3.

**Table 3:** Comparative clinical context and applicability of checkmate 743 and KEYNOTE-483 regimens

Clinical aspect	CheckMate 743 (Nivolumab + Ipilimumab)	KEYNOTE-483 (Pembrolizumab + Platinum/Pemetrexed)
Therapeutic concept	Dual immune checkpoint blockade (anti–PD-1 + anti–CTLA-4)	Chemoimmunotherapy (anti–PD-1 + platinum + pemetrexed)
Primary clinical goal	Durable, long-term disease control	Rapid tumor shrinkage and early symptom relief
Eligible population (Clinical Profile)	Younger, good PS, and immunologically “hot” tumors (non-epithelioid predominance)	Broadly applicable, including elderly or comorbid patients
Histologic subtype with notable benefit	**Non-epithelioid**: significant OS gain (HR 0.46, CheckMate 743)	**Epithelioid**: high ORR (67%, IRC-assessed) though OS not significantly prolonged
Treatment setting and accessibility	Requires inpatient monitoring in some cases due to potential immune toxicities	Widely deliverable in outpatient oncology settings
Toxicity management	Immune-related AEs (endocrine, hepatic, pneumonitis) require multidisciplinary management; generally manageable but prolonged in duration	Predominantly chemotherapy-related AEs (cytopenia, renal, GI); immune AEs less frequent and more predictable
Real-world evidence (RWE) insights	Japanese (Kanayama et al.), French (Bylicki et al.), and LATAM cohorts demonstrate reproducible outcomes across histologies; benefit extends to some epithelioid cases	KEYNOTE-A17 and JME-001 Japanese subsets show exceptional ORR (74–78%), suggesting high applicability in East Asian populations
Clinical positioning	Preferred for non-epithelioid MPM and patients seeking long-term disease stabilization	Recommended for epithelioid or symptomatic cases requiring rapid tumor reduction
Interpretative summary	Provides durable survival benefit in immunogenic subtypes; should be applied selectively based on tumor histology and patient resilience	Offers broader clinical utility with faster responses and manageable safety; promising for Japanese clinical practice context

### CQ3. Treatment options following checkmate 743 therapy

**—The role of chemotherapy after immune resistance and insights from real-world data—**.

#### Background

The CheckMate 743 trial established the clinical efficacy of dual immune checkpoint inhibition with nivolumab plus ipilimumab (Nivo + Ipi) as first-line therapy for unresectable PM. Despite this major advance, optimal treatment strategies after progression remain undefined, and no globally accepted second-line standard exists. In Japan, where approved alternatives are lacking, defining the role of chemotherapy after immune resistance has become a pressing clinical question.

#### Clinical context

Post-ICI management represents an unmet need in PM. While options such as ICI rechallenge, clinical trial enrollment, or molecularly targeted therapy are being explored, conventional platinum–pemetrexed chemotherapy has re-emerged as a practical, reimbursed, and clinically accessible choice. Evaluating its activity after prior immunotherapy exposure is essential to guide treatment sequencing and clinical decision-making.

#### Interpretation

The FLORA study (Table 4) demonstrates that platinum–pemetrexed retains meaningful antitumor activity even after exposure to Nivo + Ipi. The median PFS (approximately 5–6 months) parallels that of historical first-line chemotherapy (EMPHACIS), suggesting an absence of cross-resistance between ICI and platinum agents.

These results, though encouraging, are observational and hypothesis-generating; prospective validation remains necessary.

**Table 4:** Real-world outcomes of platinum–pemetrexed chemotherapy after Nivolumab + Ipilimumab (FLORA study [14], *n* = 277)

Histologic subtype	ORR (%)	Median PFS (months)	Median OS (months)	Key remarks
Epithelioid	42	6.0	9.5	Maintained efficacy after ICI exposure
Sarcomatoid	42	9.5	7.8	Responses in small subset
Biphasic	15	4.8	8.2	Lower activity than other subtypes
Overall	**37**	**5.6**	**8.2**	Comparable PFS to first-line EMPHACIS trial (approximately 6 mo)

ORR, objective response rate; PFS, progression-free survival; OS, overall survival

#### Clinical implications

- Efficacy Preservation: Chemotherapy efficacy appears to be preserved after prior ICI therapy, supporting its use as a second-line option.
- Patient Selection: Epithelioid and sarcomatoid histologies both demonstrate measurable benefit; however, biphasic cases show limited activity.
- Safety and Feasibility: Given its predictable toxicity and reimbursement coverage, platinum–pemetrexed remains a viable standard in Japan for patients progressing after CheckMate 743.

#### Conclusion

Following CheckMate 743 failure, platinum–based chemotherapy remains a clinically relevant and practical treatment option. Real-world data indicate that prior immunotherapy does not diminish sensitivity to platinum agents.

Future algorithms should incorporate histologic subtype, prior response pattern, and immune profile to optimize sequencing strategies.

Ultimately, the post-ICI setting represents an opportunity to refine treatment personalization and to design prospective studies integrating immunologic biomarkers for next-generation therapy development.

### CQ4. Treatment options following KEYNOTE-483 therapy

**—Optimizing post-immunotherapy strategies and ethnic considerations—**.

#### Background

In patients receiving pembrolizumab-based chemoimmunotherapy as first-line treatment in the KEYNOTE-483 trial, optimal strategies following progression remain undefined. Since ipilimumab plus nivolumab cannot be offered after prior exposure to pembrolizumab, second-line therapeutic options are extremely limited. The paucity of prospective evidence in this setting represents a major unmet clinical need in PM.

#### Clinical context and limitations

Evidence supporting ICI rechallenge or switching between PD-1 inhibitors (e.g., pembrolizumab to nivolumab) is lacking. Real-world studies in non-small cell lung cancer suggest that switching between PD-1 antibodies confers no significant clinical benefit, raising similar concerns for PM [15].

Current guidelines (e.g., NCCN Clinical Practice Guidelines 2025 [16]) do not currently recommend the use of dual ICI therapy or continuation of chemoimmunotherapy after disease progression. Therefore, treatment sequencing should be approached cautiously and clearly distinguished between established options and investigational concepts.

#### Potential post-progression strategies

Given these constraints, practical post–KEYNOTE-483 management focuses on three possible approaches:

- Reintroduction of platinum–pemetrexed chemotherapy where prior tolerance and response justify its use.
- Enrollment in clinical trials exploring novel immunotherapy combinations or targeted agents.
- Exploratory immune profiling and translational research to identify biomarkers predicting benefit from ICI rechallenge.

At present, these approaches should be framed as hypothesis-generating, not as standard practice recommendations.

#### Conclusion

After progression on pembrolizumab-based chemoimmunotherapy, there is no validated second-line regimen. Clinical trial participation and biomarker-driven research are essential to guide future sequencing strategies. Routine switching to nivolumab or dual ICI is not evidence-supported and should be avoided outside investigational contexts.

### CQ4. Supplementary

**Do Ethnic Differences Influence the Efficacy of Chemoimmunotherapy Regimens such as KEYNOTE-483?—Revisiting High Response Rates in Japanese Patients—**.

#### Background

The efficacy of immune checkpoint inhibitors (ICIs) in pleural mesothelioma (PM) has been demonstrated in global studies such as CheckMate 743 and KEYNOTE-483.

Interestingly, Japanese subset trials—KEYNOTE-A17 (a phase 1b study of pembrolizumab + platinum/pemetrexed; ORR 74%) [17] and JME-001 (a phase 2 study of nivolumab + cisplatin/pemetrexed; ORR 78%) [18]—reported substantially higher objective response rates (ORRs) than those observed in multinational studies.

In contrast, Japanese subgroup data from CheckMate 743 (nivolumab + ipilimumab) remain limited. Although real-world analyses (e.g., Kanayama et al.) have reported favorable outcomes, interindividual variability suggests that patient selection, rather than ethnicity alone, may drive these results.

Despite prior data indicating a worse prognosis for Asian patients with PM [19], both KEYNOTE-A17 and JME-001 achieved striking ORRs exceeding 70%, suggesting potential differences in treatment sensitivity that merit further investigation.

#### Balanced interpretation

The apparently higher ORRs in Japanese studies likely reflect multifactorial influences rather than intrinsic ethnic sensitivity.

Tumor burden and stage: Smaller baseline tumor volumes and earlier-stage enrollment may have enhanced chemoimmunogenic synergy.

Patient selection and toxicity tolerance: Japanese clinicians often prioritize regimens with predictable toxicity, which may favor chemoimmunotherapy over dual ICI regimens.

Host biological factors: Pharmacogenomic variability, immune gene expression, and gut microbiota diversity may modulate response, though these hypotheses remain speculative and require validation.

Recent multinational analyses provide broader context.

A real-world study from Australia (RIOMeso) reported a median OS of approximately 14.5 months for nivolumab + ipilimumab, with grade ≥ 3 toxicities around 24%, suggesting that CheckMate 743 outcomes may not be fully reproducible outside trial conditions [20].

A subsequent critical appraisal noted that, while ICIs have reshaped PM treatment, their global benefit may be more modest than initially perceived and highly dependent on patient selection [21].

Moreover, a comparative meta-analysis across three front-line RCTs demonstrated limited incremental OS benefit from ICIs after adjustment for baseline risk factors, underscoring the need for cautious interpretation of apparent regional disparities [22].

Taken together, these findings highlight the importance of viewing interethnic response variations within the context of biological heterogeneity, healthcare access, and study design differences rather than ethnicity per se.

This balanced interpretation aligns with reviewer recommendations emphasizing globally contextualized, evidence-based reasoning supported by real-world and cross-regional data.

#### Conclusion

Differences in response rates between Japanese and global cohorts should be regarded as hypothesis-generating, not confirmatory evidence of ethnic or biological divergence.

Regionally contextualized research—through Japan-based registries and translational programs—is essential to clarify whether host–tumor–microbiome interactions influence ICI efficacy.

Rather than asserting “ethnic sensitivity,” the emerging paradigm supports regionally optimized, evidence-based strategies that integrate local and global data.

As immunotherapy becomes standard in PM, interethnic and regional considerations must inform treatment optimization.

Japanese registries and RWE may reveal distinct response patterns, underscoring the importance of localized evidence to refine practice guidelines.

See Table 5 for a comparison of KEYNOTE-483, KEYNOTE-A17, and JME-001 trials.

**Table 5:** Descriptive comparison of KEYNOTE-483, KEYNOTE-A17, and JME-001

Trial	Regimen	Phase	*N*	ORR (%)	Notes
KEYNOTE-483	Pembrolizumab + platinum/pemetrexed	III	440	62 (IRC)/52 (INV)	Global; epithelioid dominant
KEYNOTE-A17	Pembrolizumab + platinum/pemetrexed	Ib	18	74	Japanese cohort; early-stage, small N
JME-001	Nivolumab + cisplatin/pemetrexed	II	20	78	Japanese cohort; single-arm
CheckMate 743	Nivolumab + ipilimumab	III	605	40	Global; higher non-epithelioid ratio

ORR, objective response rate; IRC, independent review committee; INV, investigator-assessed

#### Integrated evaluation

This clinical question re-examines potential interethnic differences in ICI-based therapy.

By juxtaposing results from KEYNOTE-483 with Japanese trials (KEYNOTE-A17 and JME-001), it provides a framework for future research on genomic, immunologic, and microbiome-derived determinants of response.

### CQ5

**Is PD-L1 expression a valid indicator for treatment selection in pleural mesothelioma?**

#### Background

PM is a histologically and immunologically heterogeneous malignancy with variable treatment responses. Although PD-L1 (programmed death-ligand 1) expression is an established predictive biomarker for immune checkpoint inhibitor (ICI) efficacy in several cancers, its clinical relevance in PM remains uncertain. Conflicting results across major ICI trials underscore the need for a cautious and evidence-based interpretation.

#### Findings from randomized trials

CheckMate 743 (nivolumab + ipilimumab vs. chemotherapy):

Among patients with PD-L1 ≥ 1%, median overall survival (OS) was 18.0 vs. 13.3 months (HR 0.69, 95% CI 0.55–0.87), while in PD-L1 < 1%, OS was 17.3 vs. 16.5 months (HR 0.94, 95% CI 0.71–1.24). No statistically significant interaction between PD-L1 status and treatment effect was observed, indicating that PD-L1 was not a robust predictive biomarker for dual ICI therapy.

KEYNOTE-483 (pembrolizumab + platinum/pemetrexed vs. chemotherapy):

Exploratory analyses from ASCO 2023 (LBA8505) showed median OS of 20.0 vs. 15.6 months (HR 0.72) in PD-L1 ≥ 1% and 15.6 vs. 13.1 months (HR 0.83) in PD-L1 < 1%. These non-significant differences suggest that chemoimmunotherapy efficacy was largely independent of PD-L1 expression. Because these data were presented at a conference and not yet peer-reviewed, they should be interpreted cautiously.

#### Evidence from prior nivolumab trials (Table 6)

Across CONFIRM [23], MERIT [24], MAPS2 [25], and NivoMes [26], no consistent association emerged between PD-L1 expression and treatment efficacy. While some small Phase II studies (e.g., MAPS2, NivoMes) reported numerically higher ORR in PD-L1–positive cases, these findings were hypothesis-generating rather than confirmatory [27]. Collectively, the data suggest that PD-L1 may carry prognostic rather than predictive significance in mesothelioma.

#### Balanced interpretation and recent evidence

Recent meta-analyses and large real-world studies [20–22] confirm that PD-L1 alone has limited predictive accuracy and should not be used to select or exclude patients from ICI-based therapy. Variability in assay platforms, scoring thresholds, and intratumoral heterogeneity likely contribute to inconsistent findings. Therefore, PD-L1 should be regarded as one component of a broader immune landscape rather than a solitary biomarker.

#### Future directions

Develop composite biomarker models integrating PD-L1 with tumor mutational burden (TMB), tumor-infiltrating lymphocytes (TILs), and immune-related gene expression signatures.

Utilize spatial and single-cell profiling to delineate immune phenotypes predictive of durable ICI response.

Incorporate clinical variables—histological subtype, tumor burden, performance status, and comorbidities—into predictive frameworks to enhance individualized decision-making.

## Overall conclusion

The therapeutic paradigm for PM has been fundamentally reshaped by the introduction of ICIs.

Landmark trials—CheckMate 743 and KEYNOTE-483—have established dual immunotherapy and chemoimmunotherapy, respectively, as first-line standards for unresectable MPM. Yet, as patient demographics, disease biology, and treatment exposure continue to diversify, new CQs have emerged in daily practice.

This review addressed five key CQs:

1. Optimal regimen selection between CheckMate 743 and KEYNOTE-483,
2. Histology-based optimization of therapeutic strategies,
3. Treatment options following CheckMate 743,
4. Management following KEYNOTE-483, including ethnic and regional considerations, and.
5. The role of PD-L1 as a predictive biomarker.

Through a synthesis of pivotal trial data, real-world evidence, and biologically informed hypotheses, we proposed a patient-centered, sequence-oriented approach to treatment selection in PM.

Importantly, rigid, “one-size-fits-all” strategies are no longer sufficient.

Optimal clinical decisions require integrated consideration of histologic subtype, tumor burden, immune contexture, patient frailty, and geographic or ethnic background. Furthermore, the development and validation of composite biomarkers—including PD-L1, TMB, TILs, and immune-related gene signatures—will be essential to advance precision immunotherapy.

As treatment options expand, biomarker-driven prospective studies and real-world registries, particularly involving Japanese and other underrepresented cohorts, will play a central role in refining future practice.

Ultimately, multidisciplinary collaboration among oncologists, pathologists, immunologists, and data scientists will be vital to translate these scientific innovations into tangible, long-term benefits for patients with PM.

## Data Availability

No datasets were generated or analysed during the current study.
